# Long-term condition management in adults with intellectual disability in primary care: a systematic review

**DOI:** 10.3399/bjgpopen18X101445

**Published:** 2018-04-21

**Authors:** Peter Hanlon, Sara MacDonald, Karen Wood, Linda Allan, Sally-Ann Cooper

**Affiliations:** 1 SCREDS Clinical Lecturer in General Practice and Primary Care, General Practice and Primary Care, Institute of Health and Wellbeing, University of Glasgow, Glasgow, UK; 2 Senior Lecturer, General Practice and Primary Care, Institute of Health and Wellbeing, University of Glasgow, Glasgow, UK; 3 Research Assistant, General Practice and Primary Care, Institute of Health and Wellbeing, University of Glasgow, Glasgow, UK; 4 Clinical Associate Professor, Health and Social Care Integration Directorate, Scottish Government, Edinburgh, UK; 5 Professor of Learning Disabilities, Mental Health and Wellbeing Research Group, Institute of Health and Wellbeing, University of Glasgow, Glasgow, UK

**Keywords:** Intellectual disability, Long-term conditions, Primary care

## Abstract

**Background:**

Adults with intellectual disabilities have higher morbidity and earlier mortality than the general population. Access to primary health care is lower, despite a higher prevalence of many long-term conditions.

**Aim:**

To synthesise the evidence for the management of long-term conditions in adults with intellectual disabilities and identify barriers and facilitators to management in primary care.

**Design & setting:**

Mixed-methods systematic review.

**Method:**

Seven electronic databases were searched to identify both quantitative and qualitative studies concerning identification and management of long-term conditions in adults with intellectual disability in primary care. Both the screening of titles, abstracts, and full texts, and the quality assessment were carried out in duplicate. Findings were combined in a narrative synthesis.

**Results:**

Fifty-two studies were identified. Adults with intellectual disabilities are less likely than the general population to receive screening and health promotion interventions. Annual health checks may improve screening, identification of health needs, and management of long-term conditions. Health checks have been implemented in various primary care contexts, but the long-term impact on outcomes has not been investigated. Qualitative findings highlighted barriers and facilitators to primary care access, communication, and disease management. Accounts of experiences of adults with intellectual disabilities reveal a dilemma between promoting self-care and ensuring access to services, while avoiding paternalistic care.

**Conclusion:**

Adults with intellectual disabilities face numerous barriers to managing long-term conditions. Reasonable adjustments, based on the experience of adults with intellectual disability, in addition to intervention such as health checks, may improve access and management, but longer-term evaluation of their effectiveness is required.

## How this fits in

Adults with intellectual disability are known to have poorer health outcomes and reduced access to health services. This review synthesises quantitative estimates of these disparities in primary care; evidence for interventions to address these; and barriers and facilitators experienced by patients and their carers. Annual health checks are effective in identifying unmet health needs but potential pitfalls exist, such as the prioritisation of Quality Outcome Framework (QOF) measures over other areas more specific to intellectual disability. This qualitative synthesis highlights important tensions, dilemmas, and solutions when attempting to facilitate access and provide primary care management for adults with intellectual disabilities, including balancing promotion of self-care while avoiding paternalistic care.

## Introduction

People with intellectual disabilities experience higher morbidity and premature mortality than the general population.^1,2^ The prevalence of multiple long-term conditions is higher, reflecting a combination of factors including genetic and biological associations with specific causes of intellectual disabilities,^3,4^ and greater exposure to environmental and social risk factors.^5^ Access to health services, screening, and health promotion is lower among people with intellectual disabilities,^6,7^ and health needs are often unrecognised or unmet.^8^ A confidential inquiry into deaths of people with intellectual disabilities in England concluded that 37% were potentially avoidable through better provision of health care.^2^ Another study reported the same rate and, in comparison with the general population, reported a hazard ratio for deaths amenable to care of 5.86 (95% confidence intervals [CI] = 5.06 to 6.80). This was despite the standard (UK Office of National Statistics) definition used not including several causes of deaths that might be amenable to care, and which were common in the people with intellectual disabilities, such as urinary tract infections and aspiration pneumonitis.^9^

Following previous calls for annual health checks and action plans,^10^ the Royal College of General Practitioners made intellectual disabilities a clinical priority in 2010. Health checks for adults with intellectual disabilities have been evaluated and introduced in several countries.^8^ Following initial development of the Comprehensive Health Assessment Programme (CHAP) in Australia, it has been implemented and adapted for other contexts.^11^ In the UK, annual health checks were introduced as a Directly Enhanced Service (DES) in Wales in 2006 and subsequently in England in 2008. While health checks may improve recognition of health needs, the long-term impact of these remains unclear.^8^ Uptake is also variable.^11^ Questions remain, therefore, about how to engage adults with intellectual disabilities and promote access to primary care; make reasonable adjustments to facilitate their care; identify and manage health needs; and improve the management of long-term health conditions.

This systematic review aims to identify and synthesise evidence concerning identification and management of long-term conditions in adults with intellectual disabilities in primary care. It aims to synthesise:

- quantitative evidence concerning identification, management, and health promotion relating to long-term conditions; and
- qualitative evidence relating to the views and experiences of patients, carers, and primary care staff to identify barriers and facilitators to the management of long-term conditions in primary care.

## Method

This review was carried out according to a pre-specified protocol.^12^ The search aimed to identify studies concerning screening, preventative health care, and long-term conditions in patients with intellectual disabilities in a primary care context. This included access to care as well as ongoing management, as both components are required to address health needs. The present authors included observational studies of healthcare use and access; trials of interventions targeting health promotion or long-term conditions; and qualitative studies examining patient, carer, or healthcare professional beliefs, attitudes, and experiences.

### Data sources

Seven electronic databases were searched using a combination of the terms 'intellectual disabilities' and 'primary care' and 'chronic disease/health promotion' (full search terms available from the authors on request). The search strategy is shown in Box 1. Inclusion and exclusion criteria are shown in Box 1.

**Box 1.** Inclusion criteria and search strategy

Criteria	Description
**Inclusion criteria**	
Population	Adults (age ≥16 years) Intellectual disabilities
Topic	Long-term health conditions Preventative health services, health checks, and screening Accessibility of primary healthcare services
Setting	Primary care
Study type	Quantitative studies Controlled trials of interventions (randomised or non-randomised) Observational studies (cohort, case control) of primary health care Analyses of routine primary care data
	Qualitative studies Qualitative interview or focus group studies Questionnaire studies
**Exclusion criteria**	
Population	Age <16 years old Without intellectual disabilities
Topic	Mental health conditions
Setting	Mental health services Secondary care or hospital setting Residential
Study type	Epidemiological studies Economic evaluations Systematic reviews Case studies or case reports
**Search strategy**	
Databases	CINAHL, Cochrane, IBSS, PsycINFO, PubMed, ScienceDirect, Web of Knowledge
Manual searching	Reference lists of all eligible studies and identified systematic reviews Hand-searching a sample of selected relevant journals (Relevant journals searched include: *Advances in Mental Health and Learning Disabilities*; *American Journal on Intellectual and Developmental Disabilities (American Journal on Mental Retardation)*; *Journal of Applied Research in Intellectual Disabilities*; *Journal of Intellectual Disability Research*; *Journal of Learning Disabilities*; *Journal of Policy and Practice in Intellectual Disabilities*; *Developmental Disabilities Research Reviews*)
Forward citations	Performed for all included studies (using Web of Science)
Restrictions	English language only
Dates	Database: January 1966 (or inception) to June 2016. Manual and forward citation search completed January 2017

### Study selection and quality appraisal

All identified titles and abstracts were screened against pre-specified inclusion criteria (Box 1). Full texts of all potentially relevant articles were obtained and assessed for eligibility. At all levels, studies were screened by two reviewers working independently, with disagreements resolved by consensus. The methodological quality of each included study was assessed using Critical Appraisal Skills Programme checklists.^13^ These varied by study design (further information available from the authors on request). Data were extracted using a pre-specified template.

### Data synthesis

Findings of included studies were combined in a narrative synthesis. Qualitative findings were synthesised using thematic analysis.^14^ The content of each study was coded according to descriptive themes, before synthesis using analytical themes based on interpretation of these findings. Findings were grouped, based on the content of eligible studies, into screening and health promotion, health checks, diabetes, and access to primary care.

## Results

**Figure 1.**
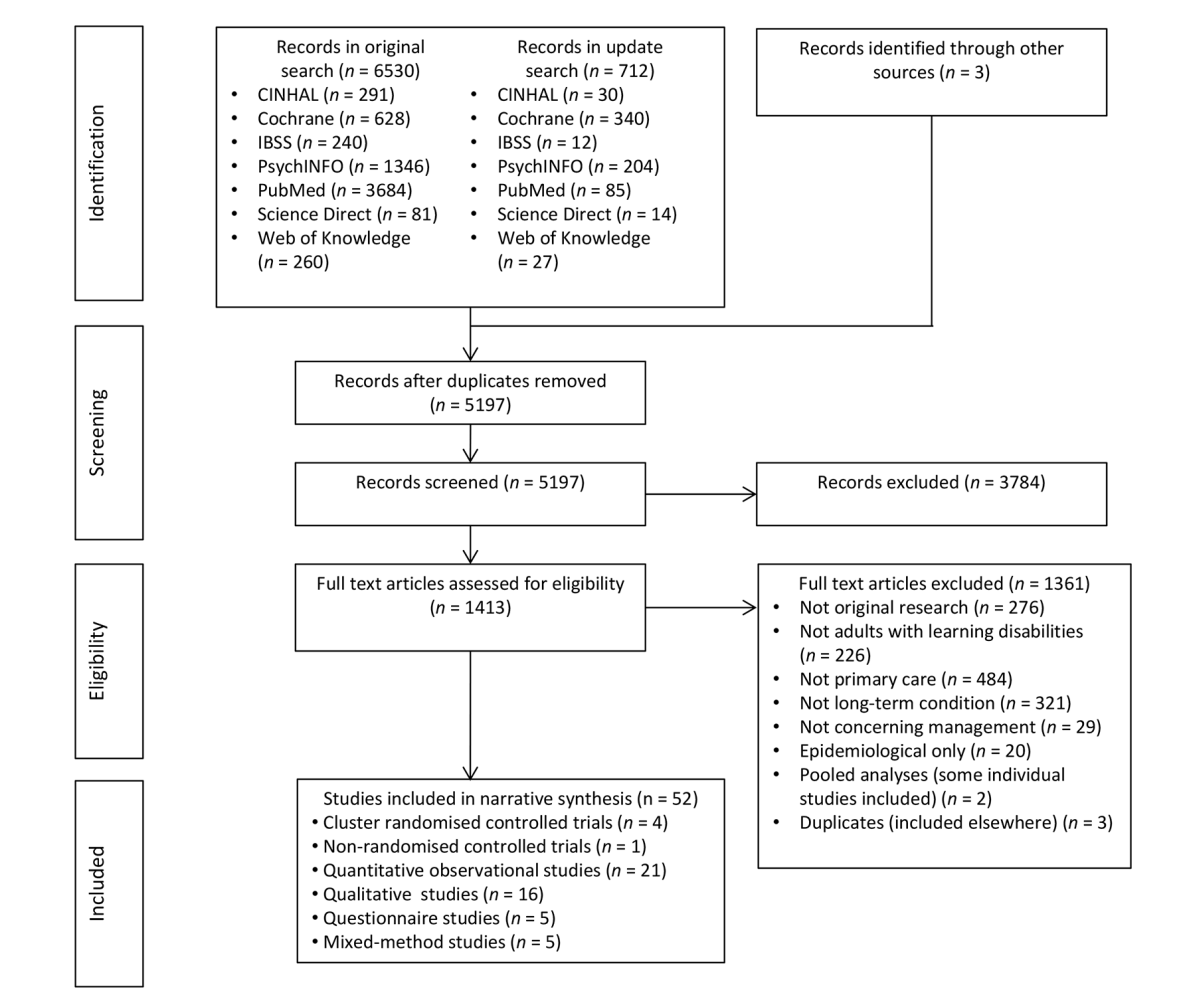
PRISMA diagram of search results.

After screening 5197 records, 52 eligible studies were identified (Figure 1).^4,15–65^ Studies were from the UK (*n* = 33), the US (*n* = 7), Australia (*n* = 4), Canada (*n* = 3), the Netherlands (*n* = 2), Taiwan (*n* = 2), and New Zealand (*n* = 1). Summaries of individual study findings are available from the authors on request.

### Screening and health promotion

Twenty-two studies concerned screening and health promotion. Cervical screening was the most widely considered. Eight studies reported lower rates of cervical screening for women with intellectual disabilities compared to women without.^15–22^ Only two of these analysed reasons for exclusion.^19,20^ Patient refusal and exclusion due to sexual inactivity were highlighted, but for many no justification for exclusion was recorded. Five surveys of healthcare professionals assessed reasons for not performing a smear, including inability of the patient to consent, communication difficulties, it being considered not in patient’s best interest,^23–26^ and sexual inactivity (although some carers felt this was an assumption).^25^ Professionals being uncomfortable performing the procedure^26^ — fearing causing distress, or being accused of assault — was also highlighted.^27^ A study of women with mild-to-moderate intellectual disabilities showed that while some had undergone cervical screening, others were 'too frightened' to do so.^28^ Interviews with intellectual disabilities nurses highlighted lack of understanding of the need for the procedure, or unfamiliarity with setting and professionals, as reasons for low uptake.^29^ A pre-post analysis of a one-to-one counselling intervention with women with intellectual disabilities showed a modest increase in uptake. In 46% of patients, a smear was deemed not in their best interest.^30^ Two randomised controlled trials of health checks showed an improvement in cervical smear uptake (considered below).^31,32^

Among those eligible, mammography,^15–17,33^ faecal occult blood,^16,17^ and prostate specific antigen^15–17^ testing was lower among adults with intellectual disabilities. Other health promotion and preventative care (such as immunisations, cardiovascular screening, and routine physical examinations) were also less commonly received by adults with intellectual disabilities.^15,17,21,22,28,34^

### Health checks

Eighteen studies concerned health checks for adults with intellectual disabilities in primary care. Eight studies, including four randomised controlled trials, demonstrated health checks leading to increased identification of previously unrecognised health needs.^19,31,32,36–41^ Identified needs included asthma, diabetes, hypertension, hypothyroidism, gastro-oesophageal reflux, epilepsy, arthritis, skin conditions, mental illness, cancer, and dementia. There was evidence that health checks led to identified needs being acted on.^31,32,36,38–40^ Two observational studies of repeated health checks suggest that additional health needs may be identified in future health checks.^37,42^ In addition, health checks resulted in increased uptake and recording of health promotion activities such as immunisations,^31,40^ blood pressure monitoring, and cancer screening.^19,31,39,43^ Practices incentivised to perform health checks carried out more tests, referrals, and medication reviews than non-incentivised practices.^44^ Despite an increase in screening uptake in some studies, overall rates remained low,^19,32 ^and others showed no increase in cervical screening, or testicular or breast examination.^38,39^

The long-term impact of health checks beyond 1 year has had limited evaluation. Evaluations of the implementation of the DES health check in England suggested the most consistent improvements in recording were in QOF-incentivised items such as disease finding and screening.^19,43^ Clinical coding of other items such as hearing or visual impairment, which are more specific to intellectual disability, was more variable.^19,43^ Controlled trials consistently demonstrate improved identification of health needs, and management of long-term conditions.^31,32,38,39^ Despite this, variable uptake and inconsistency in the recording of clinical items have led others to suggest that the model should be revised in favour of a more collaborative approach, with greater involvement of people with intellectual disabilities and their carers.^43^

Uptake of health checks in UK general practice has increased considerably following the introduction of the DES.^43,45^ However, evaluations show diverse opinions from both patients and professionals. Adults living in supported accommodation were most able to access health checks, while adults living independently sometimes feel poorly prepared.^45,46^ Some patients found the checks confusing,^47^ or experienced anxiety over tests and not understanding results.^43,47^ An evaluation of GP views following the introduction of CHAP in Australia found it to be acceptable and potentially useful.^48^ Findings from UK GPs were mixed, with some citing the lack of evidence for improved outcomes and extra workload as negatives, while others felt it encouraged appropriate use of primary care and familiarised patients and carers with available services.^47^ Patient non-attendance, low uptake, lack of staff training, and logistical difficulties in offering additional services were cited as barriers to implementation.^42,43,48–50^

### Diabetes

Six studies concerned diabetes, including four qualitative evaluations of the views and experiences of adults with intellectual disabilities and their carers.^51–54^ In two observational analyses, levels of screening for complications were lower among adults with intellectual disabilities and diabetes than national averages.^55,56^ Several reported a limited understanding of diabetes among adults with intellectual disabilities, particularly around interpreting blood glucose levels,^53,54^ the cause of diabetes,^52^ and the severity and implications of the condition.^51,52,54^ Carers reported questions were often unanswered by professionals, and paid carers highlighted a lack of training around supporting self-management, diet, or insulin therapy.^51,52^ Lifestyle modification was challenging due to incomplete understanding of dietary advice,^52,53^ and the expense of a healthy diet.^52^ Carers had an important role as a source of confidence, education, and motivation, and as an intermediary in consultations.^51–53^

### Access to and experience of health care

**Figure 2.**
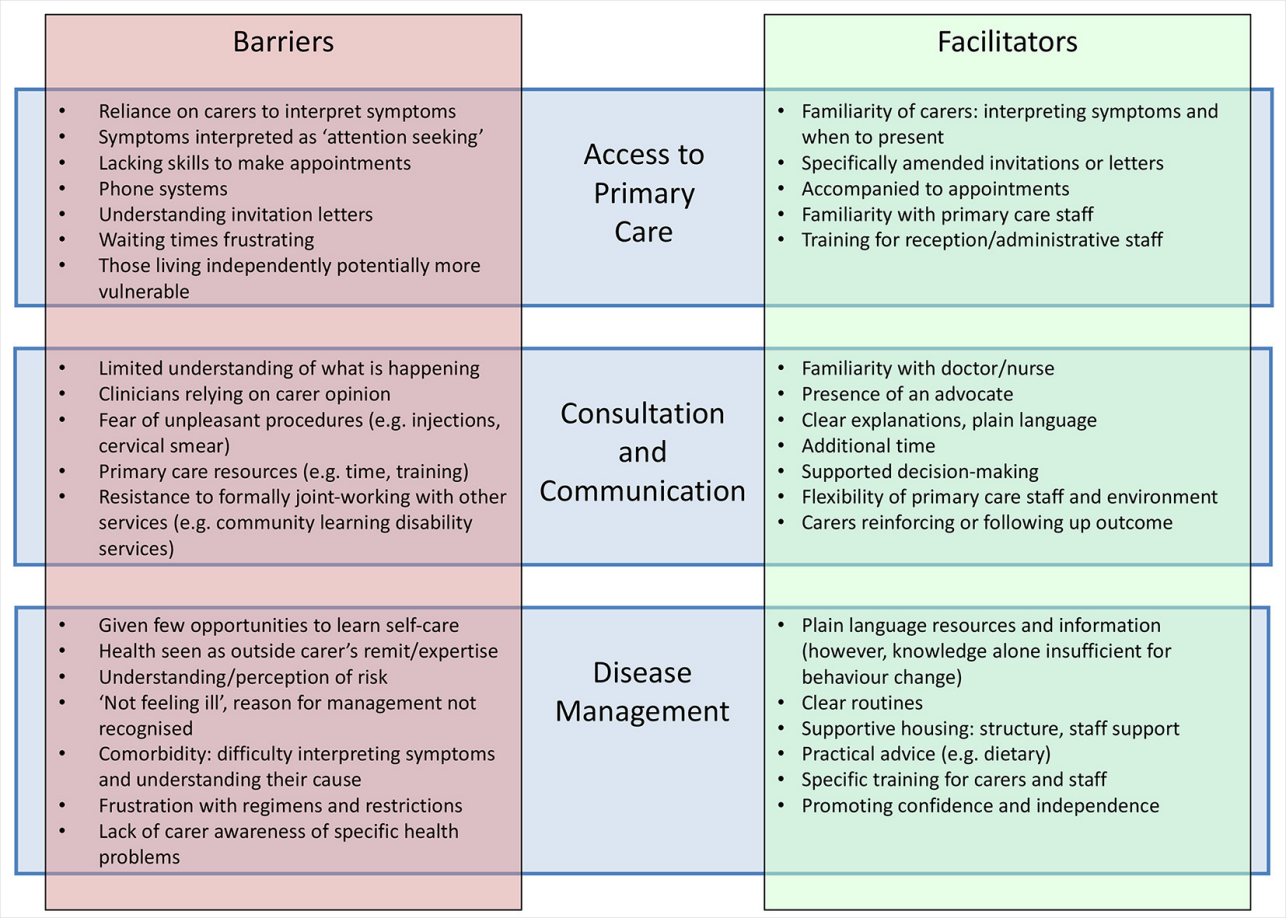
Barriers and facilitators to access and management.

An analysis of general practice data in England showed that although adults with intellectual disabilities had higher rates of chronic disease than age- and sex-matched controls, continuity of care was poorer and adults with intellectual disabilities received shorter GP appointments.^4^ Qualitative studies exploring the views and experiences of adults with intellectual disabilities,^17,28,37,46,51–53,57–59^ their carers,^25,37,51,58–60,62^ and healthcare professionals^23,24,26,27,29,57–59,61,63,64^ explored a number of difficulties experienced by people with intellectual disabilities in accessing primary care. Barriers and facilitators identified in these studies are displayed in Figure 2. Findings suggested a dilemma between promoting the independence of people with intellectual disabilities, and attempting to ensure comprehensive care for people who require support to access it. For example, individuals with milder intellectual disabilities, often living independently, were at times seen as more vulnerable and more likely not to present or to have problems overlooked. Conversely, some voiced concern about over-reliance on carer accounts when assessing problems in adults with intellectual disabilities. Many expressed a desire for clear information, supported decision making, and practical advice to improve health. Accounts of communication by healthcare professionals varied; some promoting and others hindering patients’ understanding. At other times, a limited understanding on the part of adults with intellectual disabilities, their carers, or indeed professionals, particularly of long-term illness or lifestyle risk factors, was a barrier to health promotion or disease management. In general, patients, carers, and professionals highlighted the importance of a patient-centred approach with adjustments to accommodate needs of adults with intellectual disabilities.

## Discussion

### Summary

Adults with intellectual disabilities are less likely to receive screening and health promotion than the general population. While there is some evidence that one-to-one counselling and health checks may improve uptake, overall coverage remains low. The proportion of exclusions from, for example, cervical screening that are deliberate and justified is not clear. Annual health checks can facilitate the identification of unrecognised or unmet health needs, as well as the management of long-term conditions, and have been successfully integrated into general practice in several contexts; however, their long-term impact on health-related outcomes has been little investigated. Accounts of the experiences of adults with intellectual disabilities and their carers of accessing primary care and managing long-term conditions reveal a dilemma between promoting independent self-care and ensuring access to appropriate services, while avoiding paternalistic care.

#### Findings in context

A systematic review of primary healthcare interventions for individuals with intellectual disabilities identified health checks as the only intervention showing evidence of improvements in health actions.^8^ This included five studies, all of which are included in this synthesis. The authors also highlight the need for assessment of the long-term impact of health checks. A further updated systematic review of health checks highlighted the need for future strategies to improve the cost-effectiveness and efficiency of health checks.^11^ The present review differs from these in its breadth, considering not just health checks but the management of long-term conditions in general, and includes a qualitative synthesis. The authors highlight a paucity of evidence to inform the ongoing management of long-term conditions in adults with intellectual disabilities. This qualitative synthesis demonstrates a range of experiences described by people with intellectual disabilities, and highlights the need to consider specific needs and preferences of individuals, and allow flexibility in service delivery.

National Institute for Health and Care Excellence guidelines recommend annual health checks in primary care based on the model of health checks described in this review.^65,66^ The DES in England and Wales incentivises these processes, and this review's findings suggest that implementation is feasible. However, recording is higher in items incentivised by QOF, suggesting that the wider primary care context is likely to influence how such checks are undertaken. This finding may be of particular relevance if suspension of QOF targets is considered.

### Strengths and limitations

This review synthesises a large body of literature from a range of methodologies. The inclusion of qualitative literature aids understanding of factors underlying disparities in access and uptake of health services highlighted in observational studies.^6,7^ A rigorous methodology was employed, including duplicate screening and searching of multiple sources.

The authors identified few controlled trials of interventions, and long-term follow-up data (beyond 1 year) are not available. As such, the impact on outcomes such as mortality is unknown. Disease-specific studies concerning long-term condition management were limited to diabetes. No studies were identified concerning other clinically important, long-term conditions among people with intellectual disabilities, such as epilepsy, although this may reflect service delivery, as epilepsy is often managed in secondary care learning disabilities and neurology services rather than in primary care.

The relevance of the findings of the included studies may be limited by their context. Evaluations of the implementation of health checks, or of professionals’ views, may not be directly transferrable to other healthcare settings. Social support and severity of intellectual disability is likely to influence people’s experience of accessing care, meaning qualitative findings may not be generalisable beyond their immediate contexts. This synthesis of qualitative findings was based on review of published, not original, data. Important explanatory context may therefore be overlooked.

### Implications for research and practice

These findings highlight the need to ensure adults with intellectual disabilities are not unfairly excluded from health services. Individual practices are required to make reasonable adjustments to support access of people with intellectual disabilities, and consideration of the range of identified barriers may be helpful in facilitating this process. The findings highlight the potential utility of health checks to identify problems, but also emphasise the need for longer-term evaluation of their impact. Future research, assessing the impact on outcomes such as mortality and evaluating approaches to the management of specific long-term conditions, is needed to address the health inequalities experienced by people with intellectual disabilities. This should include the development of novel interventions coupled with detailed assessment of the views, experience, and needs of adults with intellectual disability, their carers, and the professionals delivering care.
